# Prevalence and Potential Determinants of Aggregate Anthropometric Failure among Pakistani Children: Findings from a Community Health Survey

**DOI:** 10.3390/children8111010

**Published:** 2021-11-04

**Authors:** Oluwafemi Samson Balogun, Atta Muhammad Asif, Muhammad Akbar, Christophe Chesneau, Farrukh Jamal

**Affiliations:** 1 Department of Northern Europe, School of Computing, University of Eastern Finland, 70211 Kuopio, Finland; samson.balogun@uef.fi; 2 Department of Mathematics and Statistics, Faculty of Basic and Applied Sciences, International Islamic University, Islamabad 44000, Pakistan; atta.msst18@iiu.edu.pk (A.M.A.); muhammad.akbar@iiu.edu.pk (M.A.); 3 Department of Mathematics, LMNO, University of Caen, 14032 Caen, France; christophe.chesneau@unicaen.fr; 4 Department of Statistics, The Islamia University Bahawalpur, Bahawalpur 61300, Pakistan

**Keywords:** child malnutrition, CIAF, multivariate logistic regression, mother’s education, socioeconomic status, MCA

## Abstract

Malnutrition among children is an important public health problem in Pakistan. Conventional indicators (stunting, wasting and underweight) are well known. However, there is a need for aggregate indicators in this perspective. The goal of this study is to assess the prevalence and trends of malnutrition among Pakistani children under the age of five using the so-called composite index of anthropometric failure (CIAF), a tool for calculating the whole aggregate burden of malnutrition. The data were extracted from the Pakistan Demographic and Health Survey 2012–2013. Mothers’ education and socioeconomic statuses (SES) were assessed as important factors in malnutrition. Chi-squared analysis was used to check the bivariate association, and multiple logistic regression was used to identify the significant correlates of child malnutrition. Moreover, multiple correspondence analysis (MCA) was applied to strengthen the use of CIAF as an outcome variable. The study looked at 3071 children under the age of five, with 52.2% of them falling into the CIAF. Children of educated mothers had 43% fewer odds of being malnourished (OR (Odd Ratio) = 0.57, 95% CI (Confidence Interval) = 0.44–0.73). Additionally, a decreasing trend in malnutrition was found with increasing SES. There is a need to improve maternal education. Such programs focusing on increasing women’s autonomy in making home decisions should be established. Furthermore, long-term interventions for improving home SES and effective nutritional methods should be examined. For policymakers, the use of CIAF is suggested since it provides an estimate of the entire burden of undernutrition.

## 1. Introduction

The nutritional status of a population has a significant impact on a country’s socioeconomic progress. The first few years of life are crucial to a child’s mental and physical development. However, these years are set apart by micronutrient inadequacies that interferes with good growth. In addition, children of this age are not able to fight off preventable diseases [1]. As a result, nutrition is regarded as one of the most important components of the 2015 Sustainable Development Goals (SDGs). Poor nutrition can lead to an increase in the risk of infection, morbidity, and death, as well as a decline in mental development in children during their early years [2]. Additionally, it is considered that children are more vulnerable to malnutrition, and thus, child growth is an important factor in malnutrition in populations.

In 2016, 155 million children were stunted, 41 million were overweight, and 52 million were wasting, according to a joint report by UNICEF, WHO, and the World Bank group [3]. About half of these youngsters live in Pakistan, India, and Bangladesh, three South Asian countries [4]. Thus, malnutrition is a serious issue in many countries.

Pakistan is the world’s sixth most populous country and is placed it 124th out of 132 nations due to a 45% prevalence of stunting and 106th out of 130 countries due to an 11% frequency of childhood wasting [5]. In Pakistan, 0.7% of general government spending is committed to nutrition-sensitive interventions, which is lower than in other countries in the area, such as Nepal and Bangladesh, where the allocations are 3.1% and 2.1%, respectively [4].

Generally, child malnutrition is measured through conventional anthropometric indices (stunting, wasting, and underweight) in the literature [6,7]. These metrics, on the other hand, describe various aspects of malnutrition and overlap. For example, underweight is made up of both wasting and stunting, yet there is no distinction between them [8]. Despite the use of conventional indicators being frequent in the literature [9,10], some authors have pointed out that the use of these indicators could not determine the overall burden of under nutrition [11]. Therefore, there is need to use a single indicator that may capture the magnitude of nutritional status and identify the affected part of the population. According to Peter Svedberg, the use of conventional measures described above is not appropriate because they are not sufficient for the measurement of the overall prevalence of malnutrition [12]. He also claims that a new indicator that includes malnourished children is needed, and he proposes the composite index of anthropometric failure as a new measure of child nutrition (CIAF). This newly proposed index (i.e., CIAF) is based on conventional anthropometric indices through forming different failure groups (which are described in the methods section). A number of studies in the literature have used this proposed indicator (i.e., CIAF) in different parts of the world, such as [2,8,11,13,14,15] and have recommended this indicator as an alternative measure of malnutrition. However, in Pakistan, no such study has been conducted so far that can measure the prevalence of malnutrition through CIAF, as per the author’s knowledge. Therefore, this study follows Svedberg’s theory and uses this relatively robust alternative measure to examine the prevalence and association of under-five child malnutrition with other socioeconomic and demographic risk factors in Pakistan. Further, we hypothesize that CIAF gives a more accurate assessment of malnutrition among children than conventional measures.

## 2. Materials and Methods

### 2.1. Data Source

Nationally representative cross-sectional data from the Pakistan Demographic and Health Survey (PDHS) 2012-2013 were used for analysis (available at measuredhs.com (accessed on 14 August 2021)). Using a two-stage, stratified selection technique, a sample of 14,000 households was chosen. Using the probability proportional to size technique, 500 primary sampling units (252 rural and 248 urban) were selected in the first step. In the second stage, a systematic sampling process was used to choose 28 households from each primary sampling unit (a predefined number). In total, 14,569 eligible women of ages 15–49 years were selected for interview, and of these, 13,558 women were successfully interviewed. Within five years of the study, these mothers had given birth to a total of 11,763 living children. However, this analysis remains limited to 3071 live-born children of age less than 5 years with valid anthropometric information. The children whose nutritional indicators information was not present/measured were excluded from the analysis. Further details can be seen elsewhere [1].

### 2.2. Outcome Variable

The new policy-relevant anthropometric indicator of malnutrition, i.e., CIAF, is the primary outcome variable in this study. A child is termed stunted, wasted, or underweight if his or her height-for-age Z-score (HAZ), weight-for-height Z-score (WHZ), or weight-for-age Z-score (WAZ) are all less than two standard deviations from the WHO reference population median. Further details and guidelines about these growth standards can be seen elsewhere [16]. Following [17], we have categorized all children into seven groups (see Table 1 for details). A child who did not fall into any of the anthropometric failures was classified as “not malnourished” and was coded as “0”, and a child is considered to be malnourished if he/she is suffering from any of the anthropometric failure groups, and is coded as “1”. Hence, the response variable is of binary nature.

### 2.3. Explanatory Variables

A number of indicators were retrieved from the PDHS data (KR-File) and recategorized using the UNICEF conceptual framework for causes of malnutrition [18] (when this categorized operation is necessary). These indicators include regions of residence (categorized as Punjab, Sindh, Khyber Pakhtunkhaw, Blochistan, Gilgit-Baltistan, Islamabad), type of residence (rural/urban), mother’s education (categorized as no education, primary, secondary, higher), father’s education (categorized as no education, primary, secondary, higher), gender of the child (male/female), presence of other child/children of age < 5 years (yes/no), vaccination status (categorized as nonvaccinated, partially vaccinated and fully vaccinated), mother’s age at first birth (categorized as less than 20 years, 21–30 years, more than 30 years), household wealth index (categorized as poorest, poor, middle, rich, richest), type of water facility (categorized as improved and unimproved) and type of toilet facility (categorized as improved and unimproved).

The socioeconomic status (SES) (in the PDHS data this variable is labeled as wealth index (v190)) was computed by employing the principal component analysis (PCA) technique on a set of household durables (table, chair or bench, watch, radio, television, bicycle, telephone, etc.), housing characteristics such as having electricity, type of source of drinking water, access to a sanitation facility, availability of cooking fuel, main roof material, main wall material, floor material items. This proximate variable was then divided into socioeconomic quintiles: SES-I (poorest), SES-II (poor), SES-III (middle), SES-IV (rich) and SES-V (richest), based on the factor scores (PDHS report, 2013). This indicator may serve as a household wealth status and is consistent with income and expenditure measures [19]. Sahn and Stifel [20] preferred the use of an asset-based wealth index for measuring living standards and capabilities such as nutrition and health. The type of toilet facility and type of water facility were categorized following the procedure adopted by [21]. Children who have not taken even a single dose of any vaccine were considered as “nonvaccinated”, those who have taken some doses but not all, were considered as “partially vaccinated” and those who have taken all the prescribed doses of all vaccines were considered “fully vaccinated”.

### 2.4. Statistical Analysis

The statistical analysis was conducted in the following manner. First, the bivariate association between the outcome variable and different exposure variables was tested using a chi-squared test of association. Second, a multiple correspondence analysis (MCA) was performed to capture the relationship between the six categories of conventional measures (stunting (yes/no), wasting (yes/no) and underweight (yes/no). Finally, multiple logistic regression was performed to find the significant factors CIAF, which is described as follows.

Logistic regression is a popular modeling approach when the response variable is dichotomous. As the dependent variable is of dichotomous type, the possible outcomes are either “being malnourished” (taken as 1) or “being nourished” (taken as 0), therefore the magnitude of the relationships of the determinants to carrier outcomes of the children will be analyzed using the Logistic Regression models for the dependent variable “being malnourished”.

Consider a general (*k* + 1) variable equation:

$$Y_{i}^{*}=\beta^{\prime }X_{i}+U_{i}\;for\;i=1,\;2\ldots \;n$$

Here, we do not observe $Y_{i}^{*}$, instead, we observe the binary variable:

We can write it as

$Y_{i}=\{\begin{array}{c} 1\;if\;Y_{i}^{*}=\beta^{\prime }X_{i}+U_{i}>0\\ 0\;otherwise \end{array}$, Note that probability of the observed $y_{i}$ being one can be written as

$$Pr\;(y_{i}=1)=Pr\;(Y_{i}^{*}>0)$$

$$Pr\;(U_{i}>-\beta^{\prime }X_{i})=1-F\;(-\beta^{\prime }X_{i})=F\;(\beta^{\prime }X_{i})$$

If we replace *F* ($\beta^{\prime }X_{i}$) with $\wedge \left(\beta^{\prime }X_{i}\right)$

Then, we obtain the logit model. Here, $\wedge \left(\beta^{\prime }X_{i}\right)\;$ is the logistic cumulative function.

$$\wedge \left(\beta^{\prime }X_{i}\right)=\frac{e^{X^{\prime }\beta }}{1-e^{X^{\prime }\beta }}$$

In the observations, we have “*n*” cases of zeros and ones with probability of *F* *(*$\beta^{\prime }X_{i}$*)* for ones and (1 − *F (*$\beta^{\prime }X_{i}$*))* for zeros. Thus, the likelihood function is

$$Pr\;(Y_{1}=0,Y_{2}=1,\ldots \ldots \ldots ,\;Y_{n}=0)=\prod_{y_{i=0\;}}\left[1-F\left(\beta^{\prime }X_{i}\right)\;\right]\prod_{y_{i=1\;}}\left[F\left(\beta^{\prime }X_{i}\right)\right]$$

Or

$$L=\prod_{i}[{\left[1-F\left(\beta^{\prime }X_{i}\right)\right]}^{1-y_{i}}[F\;\left(\beta^{\prime }X_{i}\right){]}^{y_{i}}]$$

Taking logs on both sides, we have

$$Ln\;(L)=\sum_{i}[\;\left(1-y_{i}\right)ln(1-F\;\left(\beta^{\prime }X_{i})\right)+y_{i\;}ln\left(F\left(\beta^{\prime }X_{i})\right)\right]$$

This is the log likelihood function, and for logistic case it becomes

$$Ln\;(L)=\sum_{i}[\;\left(1-y_{i}\right)ln(1-\wedge \left(\beta^{\prime }X_{i})\right)+y_{i\;}ln\wedge \left(\beta^{\prime }X_{i}\right)]$$

Or

$$Ln\;(L)=\sum_{yi=0}ln(1-\wedge \left(\beta^{\prime }X_{i})\right)+\sum_{y_{i=1}}ln(\wedge \left(\beta^{\prime }X_{i})\right)$$

where $\beta^{’\;}$= $\left(\beta_{0\;},\;\beta_{1},\;\beta_{2},\;\ldots \ldots \ldots \beta_{k}\right)$ are the model parameters and $X^{\prime }$ = $\left(X_{0},\;X_{1},\;X_{2},\;\ldots \;\ldots \;\ldots \;X_{k}\right)$ with $X_{0}$ = 1 are the explanatory variables.

Relative odd ratios will be found to see that how the prevalence of employment varies with a unit change in a specific independent variable, keeping the remaining variables as constant for the data. Since

$$Ln\left(\;odd_{2}\right)-Ln\;(odd_{1})=Ln\left(\frac{odd_{2}}{odd_{1}}\right)=B$$

where

$$\;odd_{2}=P\left(malnourished\right)$$

and

$$odd_{1}=P\left(nourished\right)$$

$$odd_{2}/odd_{1}=exp\;(B),\;So$$

Odd−Ratio=exp (B)

Model adequacy was checked by using the Hosmer–Lemeshow test of goodness-of-fit and receiver operating characteristic (ROC) analysis. Hosmer–Lemeshow statistic follows a chi-squared distribution on (g-2) degrees of freedom, where ‘g’ denotes the number of groups (here g = 10). Following Hosmer–Lemeshow guidelines, variables were selected for multivariate analysis if they were found to be significant at a 25% level of significance in bivariate analysis. Further details about the logistic regression procedure and model validation can be seen in [22,23]. The statistical software Stata (version 14.0: Stata Corporation, College Station, TX, USA) was used for data cleansing and analysis.

## 3. Results

**Figure 1:**
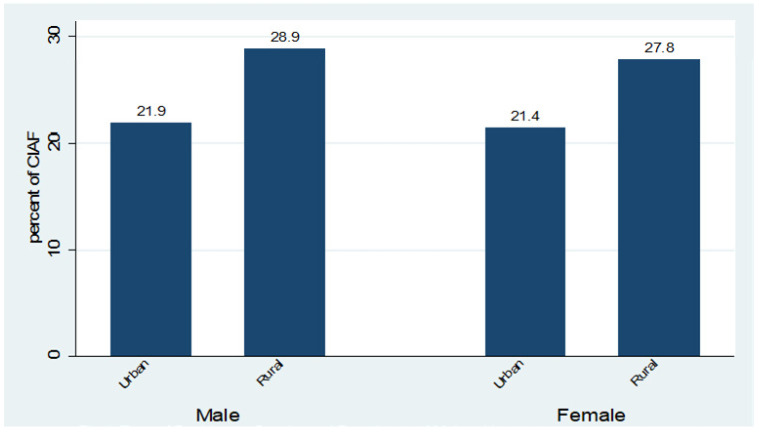
Type of Residence, Gender and Prevelance of Malnutrition.

**Figure 2:**
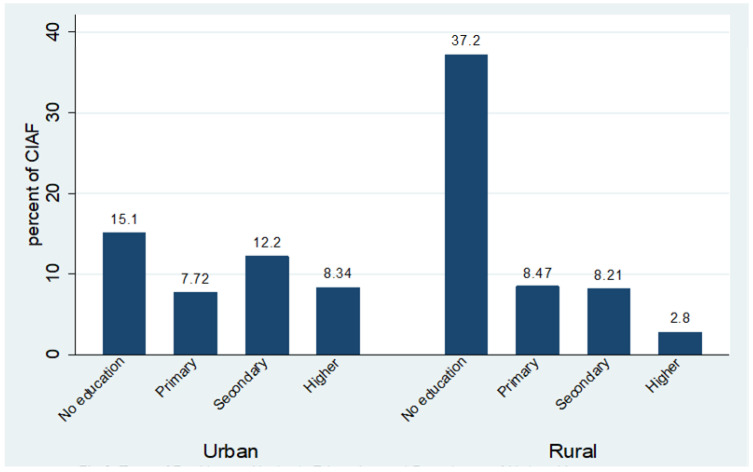
Type of Residence, Mother’s Education and Prevelance of Mulnutrition.

Characteristics of the study population: this analysis is based on data of 3071 5-year-old children. Out of these, 49.3% were female and 56.7% were living in rural areas. However, there exist disparities between rural and urban areas regarding child malnutrition. The prevalence of children falling into CIAF remained high for both male and female children in the rural areas (Figure 1). A large number of mothers were uneducated (52.3%); however, 34% of the fathers had secondary education. The prevalence of malnutrition among non-educated rural women was highest (37.2%) and this percentage shrank to 2.8% for children of educated women in rural areas. The overall position in the case of urban mothers is better. However, 15.1% of the children of urban non-educated mothers fall into the CIAF index, which is the highest among urban children (Figure 2). A major portion of the study sample contains the residents of Punjab (33.2%) and Sindh (22.9%). These regions consist of the highest burden of malnourished children (28.2% and 27.8%, respectively). Seventy-six percent of households have more than one child under five years old. A total of 634 (20.6%) households fell into the poorest wealth index (SES-I), while 19.9% of the households fell into the richest index (SES-V). Seventy-nine percent of the mothers were not working, while 50.3% were less than 20 years old at the time of their first birth. A total of 2567 (83.6%) of the households drank improved water, while 70% of the households had access to improved toilet facilities. Further details can be seen in (Table 2).

Status of malnutrition in the study population: Table 1 presents the prevalence of all groups of nutritional status. According to the conventional anthropometric measures, 45% (1380 out of 3071) of the children were stunted, 10.4% (319 out of 3071) were wasted, and 26.6% (816 out of 3071) were underweight, while 52.2% (1604 out of 3071) of the children fell into anthropometric failure. Accordingly, 4.5% of the children fall into all three and 27.1% of the children fall into a single anthropometric failure.

**Figure 3:**
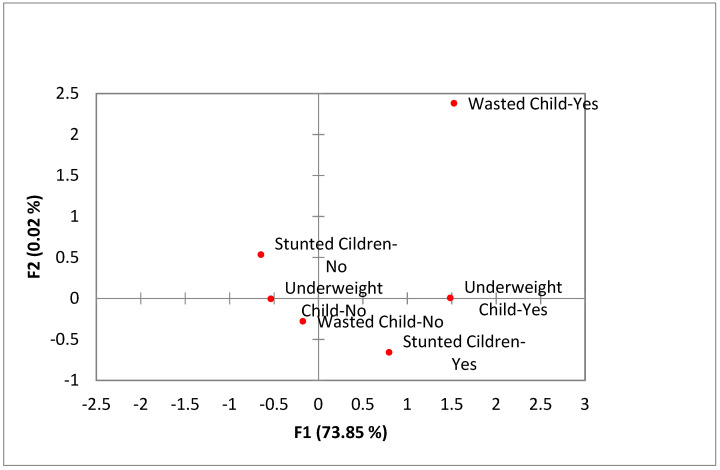
Multiple correspondence analyses of conventional measures (Stunting, wasting and underweight).

Multiple Correspondence Analyses: A total of about 74% of the inertia is explained by the first two dimensions. Points of the different categories of the same variables are clearly separated (Figure 3). Stunting and wasting appeared in the second and fourth quadrants of the plot, indicating that they are not associated. Underweight lies on the borderline between the second and fourth quarter, indicating that it is associated with both stunting and wasting, which strengthens Svedberg’s theory of using an aggregate index to measure malnutrition instead of conventional measures.

Factors associated with CIAF: The multiple logistic regression model presented in (Table 3) showed that region of residence, mother’s education, gender, mother’s age at first birth, and socioeconomic status of the household were significantly associated with the composite index. However, residence type, father’s education, vaccination, mother’s working status, and type of toilet facility had no significant association with CIAF.

Children of the Sindh region were at double the risk (OR = 1.98, 95% CI = 1.59–2.46) and children of the Baluchistan region were at about five times a higher risk (OR = 5.20, 95% CI = 3.64–7.42) of malnutrition compared to the residents of the Punjab region. Compared to uneducated mothers, the children of educated mothers (at least secondary education) had 43% lower odds of being malnourished (OR = 0.57, 95% CI = 0.44–0.73). Female children had 18% lower chances of falling within the anthropometric failure index (OR = 0.82, 95% CI = 0.70–0.95). Regarding mother’s age at first birth, children of mothers aged 21–30 years had 13% lower chances of being malnourished (OR = 0.87, 95% CI = 0.74–1.00), while for mothers aged more than 30 years, the risk of undernutrition remained about half (OR = 0.46, 95% CI = 0.24–0.84). Moreover, the children of households with higher socioeconomic status were less likely to fall within the index compared with households with lower SES (SES-V; OR = 0.39, 95% CI = 0.26–0.58, and SES-IV; OR = 0.56, 95% CI = 0.39–0.78). The results further demonstrated a rejection of the null hypothesis of the Hosmer–Lemeshow test of goodness-of-fit, indicating that the model is adequate (Hosmer–Lemeshow chi2 = 2.52; *p*-value = 0.96). Moreover, the area under the receiver operating characteristic (ROC) curve (area = 0.71) also confirmed an acceptable discrimination.

## 4. Discussions

In underdeveloped nations where the majority of the population is malnourished, assessing the nutritional status of children under the age of five is crucial [13]. According to the CIAF, the prevalence of stunting, wasting, and underweight were 45%, 11%, and 27%, respectively, while the prevalence of undernutrition was 52% among the studied sample.

According to the CIAF, the findings of this study are better than those of other countries, such as Ethiopia [8], and somewhat better than India [24]. This percentage is also high compared with the results stated in the PDHS’ (for the cycle 2012–2013) last report. This distinction might be because of the estimation strategies. However, the writings [25,26], and additionally the correspondence analysis of customary anthropometric measures, demonstrated that underweight is related to both stunting and wasting, which likewise reinforces the possibility of different investigations that CIAF provides a better gauge of children’s malnutrition compared with traditional markers [2].

There is a strong link between children’s nutritional status and their geographic location. Balochistan, followed by Sindh, is at a significant risk of falling into demographic failure. Seasonal food instability, cultural norms, and insufficient water and sanitation facilities may all contribute to this.

Different dimensions of gender discrimination have already been discussed in several research studies. Some of them demonstrated that girls are at greater risk, such as [27,28] compared to boys, while others revealed that male children are more likely to be malnourished [10,29]. However, some studies showed an absence of gender discrimination in this regard, such as [30,31]. This analysis revealed that female children have a lower chance of being malnourished, which is in line with results of other studies, such as [32] for Tanzania and [33] for West Africa. Few studies have shown that children in urban areas have better nutritional status compared with children in rural areas [14,29,34]. The outcomes of this examination uncovered no critical relationship between CIAF and any type of place of residence, which is similar to the results of some other studies, such as [35] for Bangladesh and [33] for West Africa. The mother’s age at first birth also showed a significant association with CIAF, which strengthens the hypothesis that older mothers may have better knowledge about feeding practices and other health measures of their children.

One of the key determinants of child malnutrition is parental education, especially mother’s education. Much of the literature has demonstrated that improved maternal education might be one of the defensive measures against children’s poor nutritional status [10,11,36,37,38]. This study also highlighted the fact that children of literate mothers (at least secondary education) were less likely to fall into anthropometric failure. Mother’s education may also be considered as a proxy for mother’s empowerment. A woman’s improved educational status may be considered as an increase in autonomy in family decision making. Hence, mother’s education can act as a mediator between the nutritional status of the child and decision-making autonomy [35]. Another explanation is that in poor nations, an educated mother can gain better-paying jobs and greater influence [29], which benefits the child’s health in the long term. Thus, we may conclude that the mother’s better educational level might be considered a measure of protection against childhood undernutrition. Many studies have discussed the association between household socioeconomic status and child health, such as [7,33,39].

The wealth index was used as a proxy measure of socioeconomic status to access the relationship with CIAF. It was found that the children of SES-IV and SES-V were less likely to fall into the CIAF which is in the line with other studies on the topic [7,28,29,31,40].

This may be due to the fact that children from higher-income families may have better access to good, nutritious food, whereas children from lower-income families are more likely to succumb to malnutrition due to insufficient food, high infection risk, and lack of access to basic requirements [34]. Parents from wealthy families are more likely to be educated than those from poor families, resulting in better access to food, a higher proportion of resources allocated to children’s welfare, and a higher living standard [41], all of which result in better health care for their children. Another study examining the interaction between SES and maternal education in connection to child’s health, found that the impact of better education for the mother turned out to be more defensive for the children of rich families, while the father’s education works independently of SES and is considered as a protective element [42].

The results and their interpretations of this study should, however, be considered in light of a few limitations. First, the use of cross-sectional data only allows us to investigate associations, so it is difficult to investigate cause and effect relationships. Second, the use of indirect measures of a household’s wealth status may be criticized. However, this indicator may serve as a household wealth status and is consistent with income and expenditure measures [19]. Fourth, the findings of this study rely on self-reported data, which may cause recall bias.

## 5. Conclusions

Despite the limitations mentioned above, this study has identified the most important factors influencing child malnutrition, which could have a substantial impact on the literature on the relationship between CIAF and socioeconomic and demographic characteristics. The educational condition of parents, particularly the mother’s education, must be improved. Such programs focusing on increasing women’s autonomy in making home decisions should be established. Furthermore, long-term interventions for improving home SES and effective nutritional methods should be examined.

## Figures and Tables

**Table 1:** For the creation of the response variable, different groups were used (CIAF).

Group	Characterization	Wasting	Stunting	Underweight	N (%)
A	No failure	N	N	N	1467 (47.8)
B	Wasting only	Y	N	N	92 (3.0)
C	Wasting and Underweight	Y	N	Y	88 (2.9)
D	Wasting, Stunting and Underweight	Y	Y	Y	139 (4.5)
E	Stunting and Underweight	N	Y	Y	545 (17.7)
F	Stunting only	N	Y	N	696 (22.7)
Y	Underweight only	N	N	Y	44 (1.4)

CIAF (B + C + D + E + F + Y) = 52.2%, N = No and Y = Yes.

**Table 2:** Prevalence and bivariate association for child malnutrition by exposure variables.

Exposure Variables	Child Malnourished			Chi-Squared Value
	Yes	No	Total	
	N (%)	N (%)	N (%)	
Regions				
Punjab	453 (28.2)	565 (38.51)	1018 (33.2)	213.24 *
Sindh	446 (27.8)	258 (17.6)	704 (22.9)	
Khyber Pakhtunkhaw	245 (15.3)	296 (20.2)	541 (17.6)	
Blochistan	243 (15.2)	49 (03.3)	292 (09.5)	
Gilgit Baltistan	144 (09.0)	157 (10.7)	301 (09.8)	
Islamabad	73 (04.6)	142 (09.7)	215 (07.0)	
Residence Type				
Urban	625 (39.0)	705 (48.0)	1330 (43.3)	25.80 *
Rural	979 (61.0)	762 (52.0)	1741 (56.7)	
Mother’s Education				
No Education	1003 (62.5)	603 (41.1)	1606 (52.3)	191.42 *
Primary	268 (16.7)	229 (15.6)	497 (16.2)	
Secondary	219 (13.7)	407 (27.7)	626 (20.4)	
Higher	114 (07.1)	228 (15.5)	342 (11.1)	
Father Education				
No Education	578 (36.0)	334 (22.8)	912 (29.7)	86.02 *
Primary	258 (16.1)	197 (13.4)	455 (14.8)	
Secondary	485 (30.2)	560 (38.2)	1045 (34.0)	
Higher	283 (17.6)	376 (25.6)	659 (21.5)	
Gender of Child				
Male	848 (52.9)	710 (48.4)	1558 (50.7)	6.12 *
Female	756 (47.1)	757 (51.6)	1513 (49.3)	
Vaccination				
Not vaccinated	173(10.8)	91 (06.2)	264 (08.6)	39.48 *
Partially vaccinated	909 (56.7)	763 (52.0)	1672 (54.4)	
Fully vaccinated	522 (32.5)	613 (41.8)	1135 (37.0)	
Mother’s age at first birth				
≤20 years	885 (55.2)	660 (45.0)	1545 (50.3)	39.69 *
21–30 years	701 (43.7)	764 (52.0)	1465 (47.7)	
≥31 years	18 (1.12)	43 (03.0)	61 (02.0)	
Presence of child under 5				
No	395 (24.6)	352 (24.0)	747 (24.3)	0.17
Yes	1209 (75.4)	1115 (76.0)	2324 (75.7)	
Water Quality				
Unimproved	274 (17.0)	230 (15.7)	504 (16.4)	1.10
Improved	1330 (83.0)	1237 (84.3)	2567 (83.6)	
Type of toilet facility				
Unimproved	552 (34.4)	367 (25.0)	919 (30.0)	32.26 *
Improved	1052 (65.6)	1100 (75.0)	2152 (70.0)	
Socioeconomic Status				
SES-I	431 (26.9)	203 (13.8)	634 (20.6)	193.02 *
SES-II	382 (23.8)	231 (15.7)	613 (20.0)	
SES-III	295 (18.4)	259 (17.7)	554 (18.0)	
SES-IV	293 (18.3)	365 (24.9)	658 (21.4)	
SES-V	203 (12.6)	409 (27.9)	612 (19.9)	
Working status of Mother				
Not Working	1222 (76.2)	1208 (82.3)	2430 (79.1)	17.60 *
Working	382 (23.8)	259 (17.7)	641 (20.9)	
Total	1604 (100)	1467 (100)	3071 (100)	

* represents significance at 20% level of significance.

**Table 3:** Logistic regression analysis of factors associated with child malnutrition (CIAF).

Exposure Variables	Odd Ratios (95% CI)		*p*-Value
Regions			
Punjab	1		
Sindh	1.98 **	(1.59–2.46)	<0.001
Khyber Pakhtunkhaw	0.92	(0.73–1.16)	<0.476
Blochistan	5.20 **	3.647.42)	<0.001
Gilgit Baltistan	0.81	(0.59–1.11)	<0.187
Islamabad	0.96	(0.681.34)	<0.824
Residence Type			
Urban	1		
Rural	0.95	(0.78–1.15)	<0.618
Mother’s Education			
No Education	1		
Primary	0.99	(0.78–1.25)	<0.946
Secondary	0.57 **	(0.44–0.73)	<0.001
Higher	0.59 **	(0.42–0.83)	<0.002
Father Education			
No Education	1		
Primary	0.98	(0.76–1.25)	<0.855
Secondary	0.85	(0.68–1.05)	<0.128
Higher	0.95	(0.73–1.23)	<0.700
Gender of Child			
Male	1		
Female	0.82 **	(0.70–0.95)	<0.010
Vaccination			
Not vaccinated	1		
Partially vaccinated	0.83	(0.61–1.12)	<0.227
Fully vaccinated	0.97	(0.70–1.34)	<0.868
Mother’s age at first birth			
≤20 years	1		
21–30 years	0.87	(0.74–1.03)	<0.103
≥31 years	0.46 **	(0.24–0.84)	<0.012
Working Mother			
No	1		
Yes	0.98	(0.80–1.21)	<0.87
Type of toilet facility			
Unimproved	1		
Improved	1.15	(0.93–1.40)	<0.189
Socioeconomic Status			
SES-I	1		
SES-II	1.06	(0.81–1.38)	<0.681
SES-III	0.75 *	(0.55–1.02)	<0.063
SES-IV	0.56 **	(0.39–0.78)	<0.001
SES-V	0.39 **	(0.26–0.58)	<0.001

* represents significance at 10% level of significance, ** represents significance at 5% level of significance.

## Data Availability

Data used in this manuscript can be freely accessed through “measuredhs.com” (accessed on 14 August 2021) after registration and permission.
